# Hepatocyte-like cell therapy for end-stage liver disease: From basic science to clinical application

**DOI:** 10.1016/j.livres.2026.01.005

**Published:** 2026-02-04

**Authors:** Wenwen Ge, Zhoucheng Wang, Yutong Chen, Xiao Tang, Zijian Lou, Jun Chen, Xiao Xu, Kai Wang

**Affiliations:** a Institute of Translational Medicine, Zhejiang University School of Medicine, Hangzhou, Zhejiang, China; b The Fourth School of Clinical Medicine, Zhejiang Chinese Medical University, Hangzhou, Zhejiang, China; c Hepatobiliary Center, The First Affiliated Hospital of Nanjing Medical University, Nanjing, Jiangsu, China; d Department of Hepatobiliary & Pancreatic Surgery and Minimally Invasive Surgery, Zhejiang Provincial People's Hospital (Affiliated People's Hospital), School of Clinical Medicine, Hangzhou Medical College, Hangzhou, Zhejiang, China

**Keywords:** End-stage liver disease (ESLD), Stem cell differentiation, Cell reprogramming, Cell therapy

## Abstract

End-stage liver disease (ESLD) covers the end-stage of acute and chronic liver diseases, mainly involving decompensated cirrhosis, various types of liver failure, and advanced liver cancer. Hepatocyte transplantation has shown promise in treating ESLD, but its clinical application is hampered by the shortage of donor hepatocytes. Cell therapy, an emerging effective treatment for ESLD, faces the same limitation due to scarce hepatocyte availability. Hepatocyte-like cells (HLCs), which are terminally differentiated cells, can be induced from both stem cells and somatic cells. As HLCs exhibit the morphology and function of primary hepatocytes, they offer a promising supplementary source of hepatocytes for cell therapy. First, we provide a background for the differentiation and maturation of primary hepatocytes. Subsequently, based on the current insights into the molecular pathways that regulate hepatocyte differentiation *in vivo*, we describe a strategy for establishing HLC derived from either stem cells or somatic cells. The key characteristics of these HLCs are also detailed. Furthermore, HLC offers therapeutic potential for liver failure, and HLC-based liver organoids and bioartificial liver systems have demonstrated the ability to provide liver functions, offering an innovative approach to treating various types of ESLD. Despite their promise, challenges such as efficiency in differentiation and functional maturation need to be addressed to improve the clinical application of HLCs. This review discusses these advancements and outlines the therapeutic potential and current challenges of HLC therapy for ESLD.

## Highlights

• End-stage liver disease (ESLD) involves cirrhosis, liver failure, and liver cancer, while donor hepatocytes remain scarce.
• Hepatocyte-like cells (HLCs) derived from stem or somatic cells exhibit primary hepatocyte function and morphology.
• Key molecular pathways involved in hepatocyte differentiation are leveraged to generate HLCs.
• HLC transplantation, HLC-based liver organoids, and bioartificial liver systems show promise for ESLD treatment.

## 1 Introduction

End-stage liver disease (ESLD) indicates the final stage of different liver diseases that result from acute and chronic liver injuries, such as liver failure, decompensated cirrhosis, and advanced liver cancer.^1^ Liver transplantation (LT) remains the primary therapeutic approach for ESLD. However, due to the critical shortage of donor organs, cell therapy has emerged as a supplemental therapeutic option.^2^ Hepatocyte transplantation therapy provides temporary metabolic support for patients awaiting LT. Notably, transplanted cells promote regeneration of the native liver by further colonization and proliferation. In addition, hepatocyte transplantation therapy treats patients with genetic defects in liver function in a manner analogous to gene therapy.^3^ Compared with LT, cell therapy is minimally invasive, more cost-effective, and can be administered repeatedly.^4^ Early hepatocyte transplantation studies mainly focused on primary hepatocytes (PHs), and numerous preclinical animal experiments have shown that PHs transplanted in the liver or heterotopic transplantation can survive, function, and participate in the regeneration process. Importantly, clinical PH transplantation helps patients with ESLD successfully bridge to LT.^5^

Although hepatocyte transplantation has good clinical efficacy, some problems remain intractable for the cryopreserved PHs such as cryogenic preservation damage, loss of cell phenotype after isolation, and lack of effective expansion ability *in vitro*. Due to the difficulty in culturing PHs, alternative cell sources have been explored. Human hepatocyte-like cells (HLCs) induced from stem cells or transdifferentiated from somatic cells by hepatic differentiation strategies expand the source of hepatocytes for cell therapy.^6^ The application of HLCs has largely avoided the problems of immune rejection and liver donor restriction, bringing new therapeutic strategies for the prevention and treatment of ESLD. However, the HLCs generated by current induction methods still cannot fully replicate all hepatocyte functions or match the same level of PHs. This review summarizes several established and emerging methods for HLC induction. In addition, we focused on the therapeutic role of HLC-based strategies, such as cell transplantation and bioartificial liver systems, in treating acute and chronic ESLD. In summary, HLC-based therapies offer a promising avenue for ESLD treatment.

## 2 The typical hepatocyte differentiation

Hepatogenesis goes through three stages, including definitive endoderm (DE) formation, hepatic progenitor cell formation, and hepatocyte maturation to generate complex architecture and endocrine and exocrine functions. Hepatogenesis begins around embryonic day 7, when definitive endoderm arises from the primitive streak.^7^ At embryonic day 8, fibroblast growth factor (FGF) and bone morphogenetic protein (BMP) signals from the developing heart and mesenchyme induce the specification of the competent ventral endoderm cells into the hepatocyte lineage.^8^ As the definitive endoderm forms, the cells acquire the competence to differentiate into hepatic progenitor cells through expressing the transcription factors forkhead box A (FoxA), also known as hepatocyte nuclear factor 3 (HNF3), and GATA.^9^ The hepatic progenitor cells then grow out from the ventral wall of the endoderm to form the liver bud. With the liver bud forms, hepatic lineage characteristics, including albumin (ALB**)**, alpha-fetoprotein (AFP), transthyretin, and HNF4α, are initially expressed in hepatic progenitor cells, which further differentiate into bipotential hepatic precursor cells, known as hepatoblasts. Hepatic mesenchyme growth factors, such as hepatocyte growth factor (HGF), BMP, FGF, wingless-related integration site (WNT), and transforming growth factor beta (TGFβ), continue to regulate the proliferation of hepatoblasts and the expansion of liver buds.^10^ At this stage, HNF4α plays a key role in the differentiation of hepatocyte epithelial characteristics. HNF4 deficiency leads to severe damage to liver structure and hepatocyte morphology.^11^ *In vitro*, cell differentiation without HNF4 leads to low cell maturity.^12^ During the maturation of hepatocytes, hepatic-specific genes expression undergo a dynamic and complex interregulation network consisting of self-regulatory and cross-regulatory loops. Kyrmizi and his colleagues^13^ identified six core transcription factors including HNF1α, HNF1β, FoxA2, HNF4α, HNF6, and LRH-1, which contribute to the maturation of hepatocytes. According to the embryonic development process of liver parenchymal cells, many genes and molecules have been identified and are being manipulated to promote hepatocyte culture *in vitro*.^14^

## 3 Cell sources for HLC induction

**Fig. 1:**
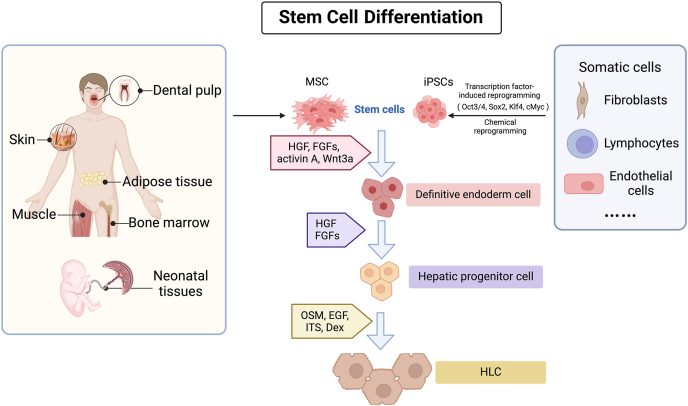
**The cell sources and differentiation process of HLC.** This figure illustrates the key stages in the differentiation of HLCs from stem cells, including MSCs and iPSCs. MSCs can be extracted from various tissues such as dental pulp, skin, adipose tissue, bone marrow, and umbilical cord. iPSCs are induced from somatic cells, such as fibroblasts, lymphocytes, and endothelial cells, through reprogramming with transcription factors including Oct3/4, Sox2, Klf4, c-Myc, or chemical induction. The differentiation process of stem cells into HLCs primarily involves three key stages: definitive endoderm cell, hepatic progenitor cell, and finally HLCs. These stages reflect the maturation of stem cells into functional HLCs capable of performing liver-specific functions. Abbreviations: c-Myc, cellular myelocytomatosis oncogene; Dex, dexamethasone; EGF, epidermal growth factor; FGFs, fibroblast growth factors; HGF, hepatocyte growth factor; HLCs, hepatocyte-like cells; iPSCs, induced pluripotent stem cells; ITS, insulin-transferrin-selenium; Klf4, Kruppel-like factor 4; MSCs, mesenchymal stem cells; Oct3/4, octamer-binding transcription factor 3/4; OSM, oncostatin M; Sox2, SRY-box transcription factor 2; Wnt3a, Wnt family member 3A.

The generation of HLCs can be roughly divided into two types: stem cell differentiation and somatic cell transdifferentiation (Fig. 1). Stem cells are considered to be an ideal cell source because of their self-renewal ability, low immunogenicity, and differentiation ability. HLCs can be derived from a variety of stem cell types, such as embryonic stem cells (ESCs), mesenchymal stem cells (MSCs), and induced pluripotent stem cells (iPSCs). However, the acquisition of ESCs carries the ethical risk of destroying embryos. Compared with ESCs, MSCs and iPSCs present fewer ethical and safety issues.^15^ MSC can be derived from various tissues such as bone marrow (BM), umbilical cord (UC), amnion, adipose tissue (AT), and dental pulp, exhibit low immunogenicity due to their low expression of major histocompatibility complex (MHC) molecules and immunomodulatory properties.^16^ The ideal and unique potential of iPSCs lies in their use in made-to-order therapies with autologous cells.^17^ Additionally, iPSCs can also minimize immune rejection by using autologous or human leukocyte antigen-matched cell lines.^18^ In recent years, stem cell-derived HLCs have emerged as a crucial candidate for treating ESLD because of their abundant availability, ease of isolation and operation, strong proliferation and differentiation capabilities, and immunoregulatory properties.^19^ Another important way of HLC induction is direct lineage reprogramming. Overexpression of hepatic lineage-specific transcription factors directly converts fibroblasts into hepatocytes.

## 4 Induction of stem cell differentiation into HLC

HLCs are generated by two major approaches: gene transduction and cytokine/small molecule-based induction.^20^^,^^21^ Gene transduction offers high efficiency and rapid induction but raises biosafety concerns due to the use of viral vectors and potential tumorigenicity (*e.g.*, c-Myc and Klf4).^22^ In contrast, cytokine- or small molecule-based protocols more closely mimic physiological hepatogenesis and avoid genetic modification, offering safer profiles for clinical application.^23^ However, they may result in less mature and heterogeneous cell populations. Understanding the advantages and limitations of these methods is crucial for optimizing HLC-based therapies.

### 4.1 Gene transduction

Hepatic differentiation protocols that utilized growth factors without gene transfer may lead to the appearance of heterogeneous hepatocyte populations.^24^ Sequential transduction of Sry-related HMG box 17 (SOX17), hematopoietically expressed homeobox (HEX), and HNF4α by adenovirus vectors in iPSCs or DE cells markedly not only enhances the endoderm differentiation but also leads to an almost homogeneous hepatocyte population.^25^ Studies have fully demonstrated that FOXA2 and HNF1α could promote efficient hepatic differentiation, making the expression profiles of hepatocyte-related genes, such as genes encoding cytochrome P450 (CYP) enzyme, coupling enzyme, hepatic transporters, and hepatic nuclear receptor, comparable to those obtained in PHs.^26^ However, the induction efficiency of MSCs to HLCs remains relatively low, posing challenges for their clinical application. Transduction of transcription factors was effective in enhancing hepatic differentiation. To avoid the risk of viral vectors and be more favorable to clinical treatment, nano-delivery systems have been exploited in HLC induction. The mesoporous silica nanoparticles (MSNs) carrying HNF3β plasmid DNA (pDNA) improve definitive hepatic induction, generate functional HLCs from iPSCs, and shorten the differentiation period within 2 weeks *in vitro*.^27^ Compared with molecule induction, gene transfer can induce HLC more efficiently but cannot avoid the risk of precise integration of transgenes.

### 4.2 Cytokine induction

**Fig. 2:**
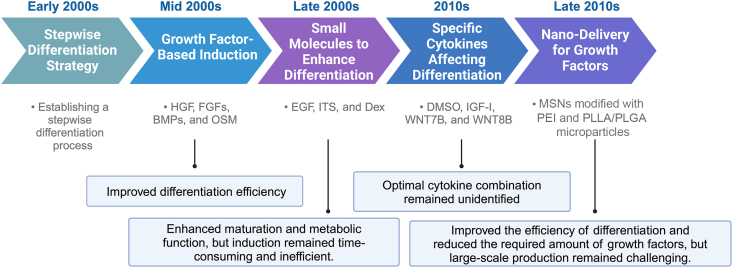
**Cytokine-induced differentiation of stem cells into hepatocyte-like cells (HLCs): a timeline of key advances.** This timeline presents the major advancements in cytokine-induced differentiation of stem cells into HLCs. It highlights key developments, including the establishment of stepwise differentiation strategies, the application of essential cytokines and growth factors (*e.g.*, HGF, FGFs, BMPs, and OSM), the incorporation of small molecules (*e.g.*, EGF, ITS, and Dex) to enhance differentiation, and the discovery of novel cytokines (*e.g.*, DMSO, IGF-I, WNT7B, and WNT8B) that improve hepatic function. Additionally, the figure showcases recent innovations in nano-delivery systems (*e.g.*, MSNs modified with PEI and PLLA/PLGA microparticles) aimed at optimizing growth factor distribution, increasing differentiation efficiency, and enhancing the functional maturity of HLCs. Abbreviations: BMPs, bone morphogenetic proteins; Dex, dexamethasone; DMSO, dimethyl sulfoxide; EGF, epidermal growth factor; FGFs, fibroblast growth factors; HGF, hepatocyte growth factor; IGF-I, insulin-like growth factor-I; ITS, insulin-transferrin-selenium; MSNs, mesoporous silica nanoparticles; OSM, oncostatin M; PEI, polyethyleneimine; PLGA, poly(DL-lactic-co-glycolic acid); PLLA, poly(L-lactic acid); WNT, wingless-related integration site.

Based on the key stages of natural liver development, a stepwise differentiation process has been established for HLCs (Fig. 2).^28^ During embryonic development, HGF elevates the expression of the endodermal marker FOXA2 during the endodermal induction when it synergizes with activin A and Wnt3a.^29^ In the first step of hepatogenesis, HGF, FGFs, and BMPs mainly promote the endodermal cells differentiating into hepatic progenitor cells, which initiates distinct phases of liver development. Further, hepatocyte differentiation medium, which typically includes factors such as oncostatin M (OSM), has been used to induce hepatoblast differentiation and maturation.^30^ Some small molecules, including epidermal growth factor (EGF), insulin-transferrin-selenium (ITS), and dexamethasone (Dex) are normally added to promote HLC-related gene expression and enhance hepatic functions. Despite the effectiveness of these factors, the overall differentiation efficiency of MSCs into HLCs remains suboptimal, necessitating further optimization of induction strategies. Notably, induction using specific signals and molecules offers a simpler, safer, and more reproducible approach, as it operates without the need for gene editing.

Some unique cytokines have been found in HLC differentiation to promote the differentiation process or improve hepatocyte functions. Alizadeh *et al*.^31^ found that dimethyl sulfoxide (DMSO) can accelerate the hepatic differentiation of AT-MSCs by facilitating transport of lipophilic and hydrophilic agents across cell membranes, making it an effective vehicle for enhancing the entrance of hepatogenic factors into stem cells. Ayatollahi *et al*.^32^ confirmed BM-MSCs treated with insulin-like growth factor-I (IGF-I) showed increased urea and ALB secretion and significant upregulation of AFP expression compared to untreated cells. Mitani and his colleagues^33^ generated HLCs with zone-specific hepatic properties with WNT signal modulators. WNT7B and WNT8B play important roles in promoting perivenous zone-specific characteristics including the enhancement of glutamine secretion, citric acid cycle, CYP1A2 metabolism, and CYP1A2 induction capacities, while WNT inhibitory factor was necessary for achieving periportal zone-specific functions such as the enhancement of urea secretion and gluconeogenesis capacities.^33^ However, the cytokines that affect HLC induction have not been identified, and the differentiation strategies are still being improved. Together, the cytokines affecting HLC induction have not been fully identified, and the differentiation strategy is still being improved.

With the deepening of research on hepatic differentiation, nano-delivery materials have been used for growth factors (GFs) delivering to promote cell differentiation more effectively. Chen *et al*.^27^ proved that MSNs modified with hyperbranched polyethyleneimine (PEI) carrying GFs can act as an efficient platform to improve engraftment of transplanted DE cells to an injured liver and promote differentiation into HLCs *in vivo*. To induce large-scale HLCs, Heidariyan *et al*.^34^ incorporated GFs-laden degradable polymeric microparticles, gelatin-coated poly(L-lactic acid)/poly(DL-lactic-co-glycolic acid) (PLLA/PLGA), within the human pluripotent stem cell spheroids for sustained release and homogenous distribution of GFs throughout the spheroids. Microparticle delivery resulted in decreased consumption of GFs compared to the conventional soluble delivery method and ensured efficient delivery of GFs to improve stem cell differentiation toward HLCs with mature liver functions.

Despite advances in HLC induction techniques, the efficiency of stem cell differentiation into HLCs remains a major limitation. Current gene transduction and cytokine-based methods have shown promise, but their efficiency requires further improvement to achieve clinically relevant levels of differentiation. The low induction efficiency of MSCs into HLCs highlights the need for optimized protocols, including more precise gene regulation, novel biomaterials for cytokine delivery, and improved culture conditions. Future research should focus on refining these strategies to enhance the practicality and therapeutic potential of MSC-derived HLCs in liver disease treatment.

## 5 Somatic cell transdifferentiation

**Fig. 3:**
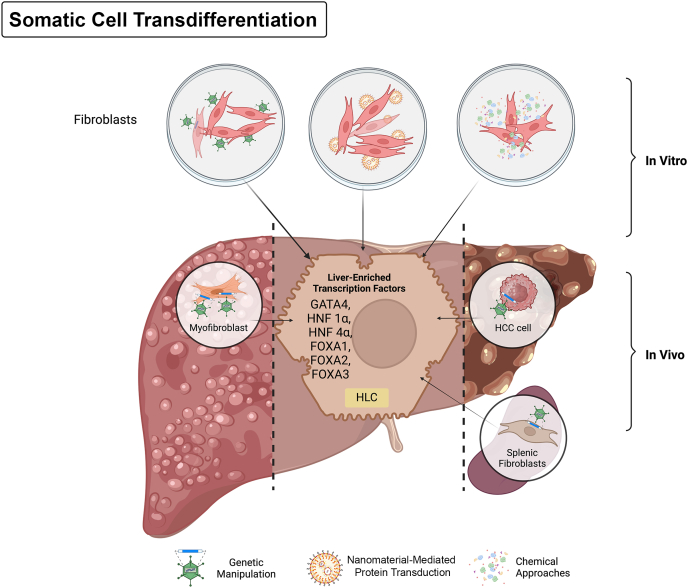
**Somatic cells transdifferentiate into hepatocyte-like cells (HLCs) by expressing liver-enriched transcription factors.** This figure illustrates the key process of somatic cell transdifferentiation into HLCs. Fibroblasts can be directly converted into HLCs *in vitro* through genetic manipulation, nanomaterial-mediated protein transduction, and chemical approaches, which activate liver-specific transcription factors such as GATA4, HNF1α, HNF4α, FOXA1, FOXA2, and FOXA3. Additionally, genetic manipulation enables the direct *in vivo* conversion of myofibroblasts, hepatocellular carcinoma (HCC) cells, and splenic fibroblasts into functional HLCs. Abbreviations: FOXA, forkhead box A; GATA4, GATA-binding protein 4; HNF, hepatocyte nuclear factor.

The induction of hepatocytes from iPSCs is a successful but complicated process. An improved technology that overexpression of lineage-specific transcription factors directly converts terminally differentiated cells into hepatic lineages has arrived (Fig. 3).

### 5.1 Genetic reprogramming

Studies have established the feasibility of generating HLCs via direct lineage reprogramming. Specific combinations of liver-enriched transcription factors—either GATA4, HNF1α, and FOXA3, or HNF4α with FOXA1, FOXA2, or FOXA3—successfully direct the conversion of mouse fibroblasts into functional hepatocytes.^20^^,^^21^ Huang *et al*.^35^ generated human HLCs from fibroblasts by lentiviral expression of FOXA3, HNF1α, and HNF4α. Direct reprogrammed cells showed an expression profile and hepatic function close to those of mature hepatocytes. However, the proliferation of transdifferentiated cells is limited, precluding them from mass expanding for *in vivo* biomedical applications. A new two-step reprogramming strategy was researched to overcome these barriers. Xie *et al*.^36^ generated expandable hepatic progenitor cells and functional human hepatocytes from fibroblasts by mimicking natural regeneration. These expandable and functionally competent HLCs are capable of replacing PHs, providing a solution to the proliferation limitation of transdifferentiated cells.

Based on the somatic cell transdifferentiation investigations *in vitro*, a few studies have begun to focus on the HLC induction *in vivo*. Cheng *et al*.^37^ reported the conversion of hepatocellular carcinoma (HCC) cells to HLCs via adenovirus-mediated delivery of HNF1A, HNF4A, and FOXA3 (Adenovirus-3Factors; Ad-3F) *in vivo*. Tumor volume in the patient-derived xenograft model treated with Ad-3F was significantly smaller than those treated with Ad-GFP.^37^ To some extent, this process could be regarded as another type of cell reprogramming, in which the disordered state of cancer cells was reprogrammed into the ordered state of hepatocytes. Similarly, *in vivo* expression of FOXA3, GATA4, HNF1α, and HNF4α via the p75 neurotrophin receptor peptide-tagged adenovirus directly converted myofibroblasts into HLCs, which can ameliorate fibrosis in damaged livers, and found that only activated fibroblasts could be effectively transformed.^38^ However, for some patients with ESLD, the damaged liver tissue environment does not even support the survival of natural hepatocytes and is not suitable for intrahepatic cell reprogramming. Ectopic regeneration offers another possible solution for ESLD. Liu *et al*.^39^ restored liver functions *in vivo* through in situ reprogramming of splenic fibroblasts into HLCs using lentiviral transfection of three key transcriptional factors (FOXA3, GATA4, and HNF1α). Different from organ or cell transplantation, this direct *in vivo* reprogramming approach could potentially lead to innovative treatments for ESLD.

These strategies avoid tumor-forming issues associated with the pluripotent cell state, but they still suffer from several shortcomings, including the residual memory of the initial cells and the limited function of target cells.^40^ The main obstacle to silencing initial cell identity genes and activating the gene regulatory networks of the target cell type may be related to the packaged H3K9me3 heterochromatin domain.^41^ Therefore, it remains necessary to develop an approach to generate desired cells that resemble their counterparts isolated from the native tissue.^36^

### 5.2 Non-genetic reprogramming

To address the low efficiency of direct reprogramming and the safety risks associated with viral vectors, lineage-specific transcription factors can be directly delivered into cells as recombinant proteins. A study demonstrated that human fibroblast-derived hepatocytes (iMPC-Heps), generated through direct reprogramming with EGF, basic FGF (bFGF), Activin A, BMP4, Dex, HGF, and OSM, efficiently repopulate mouse livers, proliferate extensively post-transplantation, and acquire functions comparable to primary adult hepatocytes, offering a promising strategy for autologous liver cell therapy.^42^ Wang *et al*.^43^ used PEI-modified silica-based nanoparticles with low toxicity as a protein-based reprogramming platform for the delivery of recombinant proteins of reprogramming factors (HNF4α/FOXA3) to mouse embryonic fibroblasts and this delivery successfully converted mouse embryonic fibroblasts into functional HLCs. This protein-based reprogramming is a promising alternative method that allows precise time and dose management and usually causes no genetic modification of target cells. Chemical approaches also have advantages over transgenic methods. Small molecules facilitate ease of manipulation, ensure standardization protocols in cell culture, and provide cost efficiency. Additionally, they do not permanently alter the genome unlike transgenic methods. Bai and his colleagues^23^ chemically induced the sequential expression of hepatocyte-enriched master transcription factors, including Hnf4α, Nr1i2, C/EBPα, and Nr1h4, using optimized chemical cocktails and culture media to reprogram mouse fibroblasts into functional chemically induced hepatocytes (CiHeps). These reprogramming methods demonstrated here do not involve targeting oncogenes, thus avoiding concerns surrounding the transplantation of genetically modified cells for therapy. However, the liver repopulation rates of CiHeps were lower than those of PHs.^23^ There are no known chemicals associated with specific cell types, and chemical reprogramming techniques are still in their early stages and require further study.

Under hepatocyte differentiation conditions, HLCs displayed hepatocytic biochemical, metabolic, physiological, and microscopic properties, including hepatic surface, transcriptional factors and structural protein expression, and the secretion of ALB, liver enzymes, bile acids, and drug transporters. Additionally, the cells exhibited lipoprotein-mediated lipid uptake and secretion, indocyanine green ingestion, glycogen synthesis, and CYP enzyme activity.^44^ Compared to human hepatocytes, most HLCs are similar to fetal-stage hepatocytes.^45^^,^^46^ It may be caused by the complex environment from fetal to adult liver development. Despite this immaturity, HLCs have shown promising therapeutic effects in animal models of liver diseases. Advances in differentiation techniques, moving from monolayer to multilayer and even multicellular cultures, have significantly enhanced the maturation of HLCs *in vitro*.^47^^,^^48^

In summary, HLC differentiation primarily relies on gene transduction, cytokine induction, and direct transdifferentiation. Gene transduction enhances differentiation efficiency but carries risks associated with viral vectors, prompting the exploration of nano-delivery systems. Cytokine induction mimics natural liver development, offering a safer and more reproducible approach, yet it remains limited by incomplete understanding of key cytokines. Direct transdifferentiation, whether genetic or non-genetic, bypasses the pluripotent state, reducing tumorigenicity but facing challenges in proliferation and functional maturity. Recent advancements, including nano-delivery of growth factors and chemical reprogramming, have improved differentiation efficiency. However, most HLCs resemble fetal-stage hepatocytes, limiting their immediate clinical application. Further research is needed to optimize maturation strategies and ensure long-term functionality for liver disease treatment.

Clinically, the choice of an appropriate HLC induction method depends on the specific therapeutic application. Gene transduction methods, despite their high efficiency, face regulatory hurdles due to safety concerns. Cytokine-based differentiation, while safer, faces challenges in generating mature and homogeneous hepatocytes suitable for transplantation. Direct transdifferentiation holds great promise for regenerative medicine, particularly in cell therapy and in situ liver regeneration. However, overcoming the current limitations in functional stability and scalability remains crucial for its clinical translation. Future research should focus on integrating biomaterials, three-dimensional culture systems, and advanced gene-editing technologies to further enhance the efficiency and clinical applicability of HLCs for liver disease treatment.

## 6 HLC transplantation therapy for ESLD

Given the clinical shortage of hepatocytes, the differentiation system meant that large quantities of HLCs could be generated for transplantation. Recent evidence supports that HLCs, which are delivered through non-surgical methods such as tail vein, intraportal, intrasplenic, and intraperitoneal injection, may alleviate liver damage by replacing injured hepatocytes, stimulating the proliferation of pre-existing ones, and reducing immunocyte infiltration or the stimulation of inflammatory cytokines, offering the potential for cell-based therapies.^49^

### 6.1 HLC transplantation

Cell transplantation for ESLD can be performed via systemic, portal, intraperitoneal, and intrasplenic injection. Evidence suggests that there is no significant difference in efficacy between intrahepatic and intrasplenic approaches.^50^ To evaluate the practical value of various HLC induction strategies, we summarized preclinical applications of gene transduction and cytokine/small molecule-based induction in representative models of liver disease, particularly focusing on acute liver injury and liver fibrosis (Table 1).^37^^,^^49^^,^^51^, ^52^, ^53^, ^54^, ^55^ In these models, HLCs restored liver function and reduced pathological damage.

**Table 1:** Preclinical studies of HLC transplantation for ESLD therapy.

Disease model	Induction methods	Key factors	Therapeutic effect	Reference
Liver failure	Direct reprogramming Gene transduction	HNF1A, HNF4A, and FOXA3	↑ ALB and survival	37
Liver failure	Stem cell differentiation Cytokine induction	EGF, bFGF, HGF, Dex, ITS, and OSM	↓ TBIL, ALT, AST, and ALP ↓ Hepatocyte necrosis area ↑ ALB and survival	51
Acute liver failure	Stem cell differentiation Cytokine induction	Activin A, ITS, FGF4, HGF, OSM, and Dex	↓ AST, ALT, and TBIL ↓ Hepatic necrosis area ↓ MDA, nitrate/nitrite, and ROS ↑ Survival	52
Acute liver failure	Stem cell differentiation Cytokine induction	Activin A, bFGF, BMP4, LY294002, CHIR99021, FGF10, HGF, and OSM	↓ AST and ALT ↑ Survival	53
Liver fibrosis	Stem cell differentiation Cytokine induction	HGF, FGF2, and nicotinamide	↓ α-SMA, ALT, and AST	49
Liver fibrosis	Direct reprogramming -Gene transduction: (type A) -Cytokine induction: (type B)	Type A: c-Myc and Klf-4 with FOXA3 and HNF4α Type B: A-83-01, CHIR-99021, BMP4, and HNF1α	↓ AST and ALT ↓ Hepatic necrosis area ↓ mRNA level of COL1α1, α-SMA, TGFβ1, and fibronectin ↓ Collagen deposition	54
Liver fibrosis	Stem cell differentiation Cytokine induction	bFGF, Activin A, FGF4, HGF, ITS, OSM, and Dex	↓ LDH and TBIL ↑ ALB	53
Acute/Chronic liver injury	Stem cell differentiation Cytokine induction	bFGF, Activin A, BMP, FGF4, HGF, EGF, and OSM	↑ ALB and survival ↓ Collagen deposition	55

Abbreviations: ALB, albumin; ALP, alkaline phosphatase; ALT, alanine aminotransferase; AST, aspartate aminotransferase; bFGF, basic fibroblast growth factor; BMP, bone morphogenetic protein; c-Myc, cellular myelocytomatosis oncogene; Dex, dexamethasone; EGF, epidermal growth factor; ESLD, end-stage liver disease; FGF, fibroblast growth factor; FOXA, forkhead box A; HGF, hepatocyte growth factor; HLC, hepatocyte-like celll; HNF, hepatocyte nuclear factor; ITS, insulin-transferrin-selenium; LDH, lactate dehydrogenase; MDA, malondialdehyde; OSM, oncostatin M; ROS, reactive oxygen species; α-SMA, alpha-smooth muscle actin; TBIL, total bilirubin; TGFβ1, transforming growth factor beta1.

In acute liver failure (ALF) mouse model, Kuo *et al*.^56^ reported the survival rate of mice increased by more than 30% after MSC-derived HLC transplantation. Fu and his colleagues^57^ rapidly induced AT-MSCs into HLCs within 10 days *in vitro*. MSC-derived HLC transplantation significantly improved survival in carbon tetrachloride (CCl_4_)-induced acute liver injury (60% *vs*. 20%) and restored alanine aminotransferase (ALT) and aspartate aminotransferase (AST) to near-normal levels. HLCs engrafted in the liver parenchyma, secreted human ALB, and replaced necrotic hepatocytes, demonstrating potential clinical application for acute liver injury treatment.^57^ Compared with the control group where all mice died, the 7-day survival rate of the UC-MSCs-derived HLCs treatment group reached 80%.^58^ Similarly, HLCs derived from human amniotic epithelial stem cells (hAESCs) improved survival to 75%.^51^

In liver fibrosis animal models, HLC transplantation reduced levels of interleukin-1beta (IL-1β), IL-6, and tumor necrosis factor-alpha (TNF-α), while upregulating nuclear factor erythroid 2-related factor 2 (NRF2) and downstream antioxidant enzymes, effectively attenuating fibrosis.^54^ Zagoura *et al*.^59^ found that molecules such as IL-10, IL-1ra, IL-13, and IL-27, secreted by HLCs, contribute significantly to liver recovery. Preclinical studies have further demonstrated that HLC therapy markedly improves survival rates in ESLD. Based on these findings, clinical investigations are gradually being carried out.

Clinically, transplantation of BM-derived HLCs has shown therapeutic benefit in patients with end-stage liver failure.^50^ Compared to standard supportive care, patients who received intrasplenic or intrahepatic injection of 1–2 × 10^6^ hepatic lineage-positive cells showed significant improvements in ascites, edema, and serum ALB from week 2. The model for end-stage liver disease (MELD) and Child scores improved significantly, with the most pronounced effect at week 2 (MELD: 11.57 to 11.12). Fatigue scale decreased from 69.1 to 54.7 at 1 month (*P* < 0.05), while performance status improved significantly. The intrahepatic group exhibited more rapid improvement in MELD score and fatigue scale, suggesting faster engraftment and functional integration of transplanted cells.^50^ More recently, a clinical trial evaluating HLCs derived from human ESCs in acute or acute-on-chronic liver failure is underway, aiming to evaluate the safety and efficacy of intravenous versus intraperitoneal delivery.^60^ HLCs transplanted into the liver could support and activate liver stem cells or progenitor cells, which then further initiate endogenous cell proliferation and differentiation. However, for patients with prolonged liver injury, multiple rounds of cell infusion may be required to achieve therapeutic benefits.

### 6.2 HLC encapsulation or HLC sheet transplantation

Bioengineering techniques have been used to promote hepatocyte engraft during cell transplantation.^61^ The first engineered hepatic graft in immunocompetent animals emerged in 2015 with human iPSC-derived HLCs by pre-engineering 3D cell co-aggregates with stromal cells followed by encapsulation in biocompatible alginate capsule formulations. Cell encapsulation followed by intraperitoneal transplantation in mice not only enables xenotransplantation in fully immunocompetent animals, but also reduces the risk of teratoma formation in future clinical applications.^62^

A study by Nagamoto *et al*.^63^ demonstrated the feasibility and therapeutic potential of human iPSC-HLC sheet transplantation on the liver surface (orthotopic transplantation) of mice with liver injury. Compared to human iPSC-HLC by intrasplenic transplantation, human iPSC-HLC sheet transplantation significantly enhanced engraftment efficiency, with human DNA fragments detected exclusively in the liver, minimizing off-target distribution. Importantly, iPSC-HLC sheet transplantation improved survival rates in mice with ALF (63.2% *vs*. 33.3% for intrasplenic transplantation and 16.7% for sham, *P* < 0.05) and effectively reduced hepatic injury, as evidenced by decreased AST and ALT levels and histological analysis.^63^ These findings highlight the advantages of HLC sheet transplantation in improving cell engraftment, liver functions, and survival outcomes, supporting its potential as an effective therapeutic strategy for severe liver diseases. Collectively, these studies outline a critical role for bioengineering techniques including enhancing the engraftment of HLCs, improving engraftment efficiency, and optimizing ectopic transplant locations, in promoting hepatocyte transplantation.

Despite the remarkable preclinical and clinical efficacy, some treatment risks still need to be considered. First, no one has been completely cured for a significant amount of time by HLC transplantation alone. HLC transplantation is often used as a means of relief during the transition period while waiting for transplantation. Second, transplanted cells release tissue factor, which activates complement and coagulation pathways. The membrane attack complexes in injected cells are triggered by complement proteins and then cells are rapidly cleared by inflammatory cells after transplantation.^64^ There is still a risk of portal vein thrombosis/celiac vascular thrombosis.^65^ Third, there is also a substantial loss (up to 70%) of these cells due to the host's immune response, known as the instant blood-mediated immune response.^66^ However, studies have shown that radiation pretreatment and serial rejection risk assessment will lead to better engraftment and long-term survival of transplanted cells.^67^ Last but not least, HLC transplantation lacks standardization, including the injected cell number, the infusion methods, and the treatment frequency. In short, a large amount of clinical evidence is still urgently needed to help large-scale clinical promotion.

## 7 Applications of HLCs in liver organoids

Single or multiple types of pre-differentiated lineage-specific cells/precursors can be aggregated and subsequently maintained in 3D culture to form spheroid or aggregate cultures. Previously studies have shown that hepatic stem cells and PHs could repopulate decellularized liver tissue. Likewise, HLCs provide an alternative source of patient-derived PHs. Human pluripotent stem cell-derived hepatic organoids were morphologically indistinguishable from adult liver tissue-derived epithelial organoids and exhibited self-renewal.^47^ Yuan *et al*.^68^ reported that encapsulated proliferating human hepatocyte liver organoids (eLO) significantly improved survival in liver failure models, ameliorated metabolic imbalances, protected gut integrity, and facilitated liver regeneration. eLO therapy was effective in both post-hepatectomy and acetaminophen-induced liver failure, with no observed toxicity or tumorigenicity, supporting its potential for clinical application.^68^ Compared to HLCs generated in 2D monolayers, 3D liver organoids exhibit higher maturity, enhanced vascularization, and improved liver-specific functions, resembling the behavior and characteristics of primary human hepatocytes and providing a valuable tool for studying and modeling complex liver activities. Kumar *et al*.^69^ has described a synthetic hydrogel-based 3D co-culture system, termed hepatocyte maturation gel, which supports the maturation and maintenance of iPSC derived hepatocytes. These liver organoids from HLCs created a new opportunity for liver disease modeling, drug discovery, disease mechanisms, and toxicology screening.

### 7.1 Disease modeling

HLCs can be generated from patients with specific liver diseases, allowing for personalized disease modeling. By reprogramming cells from patient-specific cells, such as genetic liver disorders and viral hepatitis, HLCs can mimic the disease phenotype and provide insights into disease mechanisms. This helps understand the underlying pathology, identify disease-specific biomarkers, and test potential therapeutic strategies. Complex liver diseases have recently been modeled with human iPSC-HLCs, including nonalcoholic steatohepatitis (NASH), viral hepatitis, HCC, progressive fibrosis, and cirrhosis. Ramli and colleagues^70^ utilized human iPSC-HLCs to create liver organs with hepatocytes and bile cells. Liver damage was induced in the liver organs by exposing them to a medium containing free fatty acid, and the gene expression between liver tissue from individuals with NASH and liver-damaged organs was similar, suggesting that the produced liver organs may be able to replicate NASH *in vitro*. Studies have demonstrated hepatitis B virus (HBV)-induced innate immune responses in HLCs and liver organoids derived from human iPSCs, and iPSC-HLCs can support long-term HBV infection.^71^, ^72^, ^73^ Liu *et al*.^74^ induced the differentiation of human ESCs into adult hepatocytes in order to investigate the oncofetal properties of primary tumor tissues. Moreover, disease models using human HLCs from patients might help identify the key genes and regulators associated with donor susceptibility, which is conducive to developing novel individualized treatments.

### 7.2 Drug screening

Liver diseases often lack effective treatments, and testing potential drugs on patient-specific iPSC-derived HLCs can help predict drug efficacy and toxicity. This approach identifies drugs more likely to be effective in specific patient populations. Takayama *et al*.^75^ demonstrated that HLC spheroids created from 3D iPSC-derived HLC progeny displayed greater CYP450 enzyme activity compared with HLCs maintained in 2D and that the HLC spheroids could more accurately detect hepatotoxic drugs compared to 2D HepG2 spheroids. Chen *et al*.^76^ found the drug metabolism potential (Cyp3a11 and Cyp1a2) of HLCs was greatly enhanced and reached levels that were close to PHs when HLCs repopulated into decellularized liver tissue, resulting in a liver model that is more representative of the native liver tissue and has comparable hepatic features. The system of HLCs-on-decellularized-liver-tissue could serve as an excellent model for conducting phase I drug metabolism studies. Ghosh *et al*.^77^ utilized human iPSC-derived HLCs to classify hepatotoxic and non-hepatotoxic chemicals and conducted mechanistic toxicity studies. The iPSC-derived HLCs accurately identified chemicals that cause liver injury, and TempO-Seq technology linked cytotoxicity to oxidative stress and unfolded protein response pathways through transcriptomics data on treated HLCs. iPSC-derived HLCs generated by overexpressing transcription factors in the optimized medium are suitable for chemical toxicity detection and mechanistic toxicity studies.^77^

Despite the ongoing improvements in organoid platforms, it remains challenging to cultivate multiple cell types on a single platform. While these improvements have led to better physiological interactions between different systems, such as the immune and vascular systems, the size of organoids produced through this method is limited and still a simplified version of the complex architecture and cellular diversity found in native tissue.^78^, ^79^, ^80^ Additionally, replicating the cellular diversity of the liver is a costly and time-consuming process. Overall, iPSC-derived HLCs in 3D liver models provide a powerful tool for studying liver biology, disease mechanisms, and drug development. In the future, we expect to apply 3D bioprinting technology to replicate functional organs with tissue structure using HLCs and scaffolds in order to more accurately simulate natural tissues and show better therapeutic effects in disease treatment.^78^

## 8 Application of HLCs in bioartificial liver (BAL) support system

BAL support system has been developed in the last few decades to bridge LT or to facilitate liver regeneration to prevent severe complications caused by ESLD and improve survival. BAL devices have cell-housing bioreactor containing hepatocytes to provide biotransformation and replace the most important liver functions, including oxidative detoxification, biotransformation, excretion, and synthesis.^81^ Previous studies have shown that implanting BAL support system with primary porcine hepatocytes is effective for treating ALF.^82^ However, the PHs cannot meet the cell number requirements and the porcine hepatocytes have risks in immunogenic reactions and xenozoonosis, making them unsuitable for use in BAL devices.^81^^,^^83^ FOXA3, HNF1A, and HNF4A-induced HLCs with the functional characteristics of hepatocytes are expandable cells, which allows for the stable production of functional hepatocytes in large quantities and well adapted for application with the BAL system and future ESLD therapy.^35^ It has been proved that HLCs can maintain viability, epithelial morphology, and liver gene expression after treatment with bioreactor medium. Shi *et al*.^84^ obtained about 3 billion cells within 7 day and applied the expanded human HLCs in a homemade BAL support system (hHLC-BAL). The ALT, AST, ammonia, total bilirubin (TBIL) levels, and prothrombin time were already corrected in HLC-BAL group within 7 days, and survival was improved by 87.5% compared with BAL without functional cells group.^84^ In another major study, Chen and his colleagues^85^ reported the BAL device assembled with human iPSC-derived hepatic spheroids, which expressed important mature hepatic genes at comparable levels to PHs, rescued the ALF pigs. This BAL device was able to restore ALT, AST, ammonia, and TBIL levels to healthy levels in just 7 days and maintained subsequently.^85^ The latest research by Wang *et al*.^86^ reported a clinical-grade BAL device employing human HLCs manufactured under good manufacturing practice conditions. In the porcine post-hepatectomy liver failure model, the hHLC-BAL device treatment restored the remnant liver's functions, specifically ammonia detoxification, and facilitated liver regeneration, showing a remarkable survival benefit.^86^ Following the experimental of large animals, seven patients who underwent extended liver resection were treated with hHLC-BAL support system. Patients with hHLC-BAL treatment not only showed improved liver function and enhanced liver regeneration, but also demonstrated good tolerability, meeting the primary outcomes of safety and feasibility.^86^ However, the trial lacked control for HLC in the large-animal study. Considering all of this evidence, it seems that the unlimited proliferation feature of HLCs enables the creation of an off-the-shelf hepatocyte bank for the production of functional hepatocytes in large quantities, which are well adapted for application with the BAL system and future ESLD therapy.

## 9 Application prospect of humanized chimeric liver with HLC

Chimeric animals contain cells from different species. The humanized chimeric liver replicates the structure of the human liver and performs important human-specific metabolic/homeostasis processes and could be used as the disease model for pathological and pharmaceutical research.^87^ In a new study, the authors developed a novel chimeric mouse liver, repopulated with rat hepatocyte, to study efficacy of the transplanting rat-to-mouse chimeric livers into rats.^88^ They found that after transplant, the chimeric grafts underwent post-transplant remodeling with rat hepatocytes replacing all mouse hepatocytes within 56 days and led to long-term survival in rats with the use of suboptimal immunosuppression. In addition, the mouse chimeric grafts grew up to 8.6 times their original size, which is more than the rat control grafts.^88^ The use of chimeric livers offers an advantage over classical xenotransplantation due to the production of autologous proteins for the recipient. It has been reported that a robust human liver chimeric animal model was developed by engrafting the human iPSC-HLCs to *Fah*^*−/−*^*Rag2*^*−/−*^*IL-2Rγc*^*−/−*^ SCID (FRGS) mice.^73^ After being optimized by a small molecule, XMU-MP-1, the human iPSC-HLCs engrafted FRGS (HLC-FRGS) mice exhibited approximately 40% liver chimerism at week 6 after engraftment and maintained at this level for at least 14 weeks. Furthermore, the human HLCs survived in the mouse liver for a long time (over 20 weeks) and showed stable human liver functions and no evidence of tumorigenesis in the main organs.^73^ In another major study, the administration of an agonist c-Met receptor antibody *in vivo* greatly enhanced the expansion of implanted human iPSC-HLCs in fumarylacetoacetate hydrolase deficient mice.^89^ This resulted in a significant increase in human ALB levels and high human liver chimerism (over 40%) in the transplanted mice at week 8 after transplantation.^89^ The evidence presented in this section suggests that human HLCs have the potential to replace the PHs and form functional chimeric livers that may solve the major limitation in the field of LT. However, the present data represent the first step towards a potential clinical application, and studies about humanized chimeric livers in large animals, including pigs, rhesus macaques, or dogs, are necessary for further research to obtain replacement organs for clinical LT.

## 10 Conclusions and perspectives

In summary, HLCs can be generated reproducibly and effectively from MSCs, iPSCs, and somatic cells. A review of several available reprogramming approaches suggests that there are multiple pathways to induce HLCs, including gene-based reprogramming, protein-based reprogramming, and chemical induction.

Although HLCs express several liver-specific markers and demonstrate partial functional capabilities, they consistently fall short of fully recapitulating the mature phenotype of adult primary hepatocytes. This immaturity limits their application in disease modeling, drug screening, and cell therapy. One major contributing factor is abnormal transcriptional regulation. Incomplete or unbalanced expression of key transcription factors such as HNF4α, FOXA2, and C/EBPα impairs the establishment of mature liver-specific gene regulatory networks.^90^ Moreover, epigenetic barriers (*e.g.*, persistent histone modifications and DNA methylation at liver-specific loci) may also hinder complete lineage commitment.^91^ Another major contributor to this immaturity is the absence of heterotypic cell-cell interactions, which are normally present between hepatocytes and non-parenchymal cells in the liver. These interactions, within a three-dimensional microenvironment, are critical for hepatocyte polarization, function, and regeneration.^92^ Conventional HLC differentiation protocols fail to replicate this complex multicellular architecture, resulting in incomplete maturation. Within this context, engraftment into the native liver parenchyma is believed to provide essential microenvironmental cues that can promote further maturation and long-term repopulation of HLCs.^93^ Bridging this gap will require improvements in both intrinsic cellular programming (*e.g.*, through modulation of key transcriptional networks) and extrinsic environmental factors, such as 3D co-culture systems, organoid-based engineering, and *in vivo* pre-conditioning of the host liver. Understanding and manipulating these limiting factors will be essential to enhance the therapeutic efficacy and experimental applicability of HLCs.

**Fig. 4:**
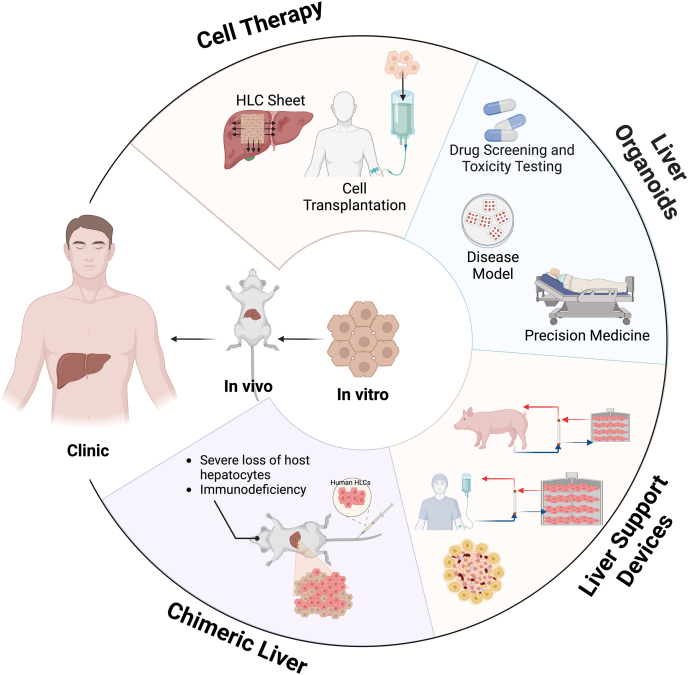
**The application of HLCs in cell transplantation, liver organoids, bioartificial liver support system, and chimeric liver.** This figure illustrates the preclinical and clinical applications of HLCs, including cell transplantation, liver organoids, bioartificial liver support systems, and chimeric liver. HLC transplantation has been explored as a potential therapeutic approach for liver regeneration and function restoration. Liver organoids derived from HLCs serve as *in vitro* models for disease modeling and drug screening. The integration of HLCs into bioartificial liver support systems aims to provide temporary liver function support for patients with liver failure. Additionally, chimeric liver approaches, utilizing HLCs in engineered liver tissues, represent a novel strategy for liver regeneration and transplantation. Abbreviation: HLCs, hepatocyte-like cells.

Additionally, the lack of standardization in the morphological, phenotypic, and functional characteristics of HLCs makes challenges for comparison between published studies. In this review, we also focus on the applications of HLCs. Their ability to mimic hepatocyte functions makes them valuable tools for improving clinical approaches for ESLD (Fig. 4). In preclinical studies, HLC transplantation has shown promise in alleviating liver injury and improving prognosis of ALF, liver fibrosis, and other liver diseases. However, treating chronic ESLD remains a challenge, though multiple transplantations of HLCs may offer the potential therapy. Compared to two-dimensional cell cultures, three-dimensional hepatic organoids derived from HLCs exhibit higher maturity, vascularization, and liver-specific functions, resembling the function and characteristics of PHs. This provides valuable tools for studying and simulating complex liver activities. Furthermore, as HLCs can be generated from patients' MSCs and fibroblasts, we can establish personalized liver disease models, offering a valuable approach to personalized medicine. With the advances in the capability of stable production and lacking xenogeneic immunogenicity, HLCs serve as an excellent cellular source for BAL devices to provide liver function replacement. Furthermore, the development of functional human chimeric livers using HLCs is crucial for solving the transplantation dilemma in the future. Future research should focus on promoting the functional maturity of HLCs and improving their engraftment efficiency. Refining differentiation protocols, optimizing three-dimensional culture conditions, co-culturing with non-parenchymal liver cells, and incorporating biomaterial scaffolds may enhance their hepatic functions. Long-term engraftment stability remains another key challenge, requiring further exploration of transplantation strategies and immune evasion techniques. Standardization of HLC characterization is also crucial to ensure reproducibility and comparability across studies. Preclinical validation in large animal models with long-term follow-up will be essential to assess the safety, integration, and therapeutic efficacy of HLC-based therapies. Additionally, innovative genetic and cellular engineering approaches, such as CRISPR-mediated gene editing, could further enhance HLC functionality while minimizing immunogenicity. By addressing these challenges, HLC research can progress toward clinically viable applications, offering novel therapeutic strategies for ESLD and other hepatic disorders.

## Authors' contributions

**Wenwen Ge:** Writing – review & editing, Writing – original draft, Conceptualization. **Zhoucheng Wang:** Writing – original draft, Visualization, Conceptualization. **Yutong Chen:** Writing – original draft, Visualization. **Xiao Tang:** Writing – original draft. **Zijian Lou:** Writing – original draft, Visualization. **Jun Chen:** Supervision, Funding acquisition. **Xiao Xu:** Project administration, Funding acquisition, Conceptualization. **Kai Wang:** Writing – review & editing, Supervision, Project administration, Funding acquisition, Conceptualization.

## Declaration of competing interest

The authors declare that there is no conflicts of interest.
